# Angle-Matched Isometric and Isokinetic Hamstring-to-Quadriceps Ratios Are Not Directly Interchangeable: An Exploratory Multi-Angle Dynamometry Study

**DOI:** 10.3390/bioengineering13070744

**Published:** 2026-06-26

**Authors:** Zhaoxiang Zhang, Patrycja Bocian, Piotr Aschenbrenner

**Affiliations:** 1 Doctoral School, Gdańsk University of Physical Education and Sport, Kazimierza Górskiego Street 1, 80-336 Gdańsk, Poland; patrycjabocian3@gmail.com; 2 Faculty of Physical Culture, Gdańsk University of Physical Education and Sport, Kazimierza Górskiego Street 1, 80-336 Gdańsk, Poland; piotr.aschenbrenner@awf.gda.pl

**Keywords:** hamstring-to-quadriceps ratio, isometric contraction, isokinetic dynamometry, angle-specific torque, knee joint, muscle strength, repeated-measures analysis

## Abstract

Hamstring-to-quadriceps (H/Q) strength ratios describe knee flexor-to-extensor torque balance, but their interpretation may depend on joint angle, contraction mode, and denominator stability. This exploratory repeated-measures study examined whether angle-matched H/Q ratios obtained from isometric and isokinetic dynamometry are directly interchangeable. Fourteen healthy young adults performed maximal knee extension and flexion using a Biodex System 4 Pro. Isometric testing was conducted at 10°, 30°, 60°, 90°, and 110° of knee flexion; isokinetic testing was performed at 60°/s, 150°/s, and 300°/s, with torque extracted at matched angles. H/Q ratios were calculated as flexor torque divided by extensor torque for each angle, limb, and mode and analyzed after log transformation. In the all-angle model, log-transformed H/Q ratio showed a significant angle effect (F(4,52) = 14.60, *p* < 0.001, partial η^2^ = 0.529), limb effect (F(1,13) = 5.54, *p* = 0.035, partial η^2^ = 0.299), angle × mode interaction (F(12,156) = 2.78, *p* = 0.002, partial η^2^ = 0.176), and angle × mode × limb interaction (F(12,156) = 2.60, *p* = 0.004, partial η^2^ = 0.167). After excluding endpoint angles, the angle × mode interaction remained significant (F(6,78) = 8.60, *p* < 0.001, partial η^2^ = 0.398). These findings suggest that angle-specific H/Q ratios are influenced by joint position and testing mode. Because of the small sample and absence of protocol-specific test-retest reliability, the results should be interpreted as exploratory and hypothesis-generating. Isometric and isokinetic H/Q ratios should not be treated as directly interchangeable, and endpoint ratios should be interpreted together with the underlying flexor and extensor torque values.

## 1. Introduction

Muscle force production is strongly influenced by muscle length and contraction velocity, which are classically described through the force-length and force-velocity relationships. In applied sports science, rehabilitation, and clinical biomechanics, these mechanical principles are commonly assessed using isometric and isokinetic dynamometry [1,2,3,4]. Although range of motion does not itself determine muscle force, it defines the joint-angle span across which muscle length, moment arm geometry, and neuromuscular control interact to influence net torque production.

Isometric testing quantifies force production at a fixed joint angle and is frequently used because it is relatively simple, safe, and reliable when familiarization, positioning, stabilization, gravity correction, and reporting procedures are standardized [5,6,7,8,9,10,11]. However, isometric strength is angle-specific; values obtained at one joint position may not represent strength across the full movement range [5,6,7]. Multi-angle isometric testing has therefore been proposed as a more complete approach for characterizing joint-angle-dependent strength properties. Isokinetic testing, in contrast, evaluates torque during movement at a controlled angular velocity and can provide angle-specific information across a continuous range of motion [3,4,9,10]. Previous studies have reported associations between isometric and isokinetic peak torque values, but the two modes remain mechanically distinct because they impose different constraints on joint movement, contraction velocity, stabilization, and torque production.

The hamstring-to-quadriceps (H/Q) ratio is one of the most common outcomes derived from knee dynamometry. It is typically used as a descriptive index of knee flexor and extensor strength balance, and its interpretation depends on whether the ratio is conventional, functional, mixed, peak-torque based, or angle-specific [12,13,14,15,16]. At the same time, prospective studies and systematic reviews have cautioned against interpreting the H/Q ratio as a simple or isolated predictor of hamstring or anterior cruciate ligament injury risk [17,18,19]. A major limitation of conventional H/Q assessment is that ratios are often based on peak torques that occur at different joint angles, or they are interpreted without sufficient attention to contraction mode and joint position [12,13,14,15,20,21]. Because the quadriceps and hamstrings have different torque-angle profiles, a ratio calculated at one angle can convey a different meaning from a ratio calculated at another angle.

This methodological issue is particularly important when isometric and isokinetic values are compared. In practice, H/Q ratios derived from different testing modes are sometimes discussed as though they represent the same construct. However, angle-specific analyses in athletes and anterior cruciate ligament-deficient or reconstructed populations show that torque deficits and H/Q-ratio differences can depend on the joint angle at which they are assessed, and may not be fully captured by peak-torque metrics alone [20,21,22,23,24,25,26]. Existing angle-specific studies have mainly emphasized isokinetic torque curves, ACL-related deficits, normative sport-specific profiles, or clinical recovery after anterior cruciate ligament injury [20,21,22,23,24,25,26]. Less is known about whether an H/Q ratio calculated at the same knee angle has the same interpretation when obtained from a fixed-position isometric contraction versus a moving isokinetic contraction. This distinction is relevant because the same flexor/extensor equation may combine different denominator behavior, contraction velocity, and range-of-motion constraints across modes.

Therefore, the purpose of the present exploratory study was to examine angle-specific H/Q ratios under isometric and isokinetic testing conditions at matched knee joint angles. The primary outcome was the log-transformed angle-specific H/Q ratio. The primary analysis examined the within-participant effects of joint angle, contraction mode, and limb. Secondary analyses evaluated endpoint behavior, central-angle sensitivity, and selected angle-by-limb post-hoc contraction-mode comparisons. We hypothesized that H/Q ratios would be angle-dependent and that the relationship between contraction mode and H/Q ratio would differ across joint angles. Because of the small sample size, all findings were treated as exploratory and intended to inform future reliability, validity, and longitudinal studies rather than to establish clinical cut-off values or injury-risk prediction.

## 2. Materials and Methods

### 2.1. Participants

Fourteen healthy young adults (five females and nine males) volunteered to participate in the study. Participants had a mean ± SD age of 21.36 ± 2.90 years, height of 173.79 ± 6.24 cm, and body mass of 70.93 ± 9.09 kg (Table 1). Participants were recruited from the Gdańsk University of Physical Education and Sport and reported no current lower-limb musculoskeletal injury at the time of testing. Reported sport or physical-activity backgrounds were fitness (*n* = 7), running (*n* = 2), dance (*n* = 1), gymnastics (*n* = 1), kickboxing (*n* = 1), karate (*n* = 1), and swimming (*n* = 1). Dominant limb was recorded before testing and coded as right in eight participants and left in six participants. Detailed training volume, years of sport participation, and competition level were not used as analytic variables because they were not recorded with a validated training-status instrument. Therefore, sport background and limb dominance were reported descriptively and were not used for inferential subgroup analysis. Written informed consent was obtained from all participants before data collection.

No a priori sample-size calculation was performed before data collection. The study was therefore treated as an exploratory repeated-measures methodological analysis rather than a confirmatory trial. The available sample provided 560 condition-level observations (14 participants × 2 limbs × 4 modes × 5 angles), but the number of statistically independent participants remained 14. Accordingly, statistical inference was interpreted using exact test statistics, effect sizes, approximate 95% confidence intervals for partial eta squared, and sensitivity analyses, with emphasis on the consistency of within-participant patterns rather than on isolated pairwise *p*-values. The sample is not sufficient to establish clinical thresholds, injury-risk prediction, or subgroup effects by sex, sport, or limb dominance.

**Table 1:** Demographic characteristics of participants.

Parameter (N = 14)	Minimum	Maximum	Mean	SD
Age (years)	19	30	21.36	2.90
Height (cm)	162	186	173.79	6.24
Body mass (kg)	55	82	70.93	9.09

### 2.2. Experimental Design and Testing Procedures

Participants completed knee extensor and flexor strength assessments using a Biodex System 4 Pro isokinetic dynamometer (Biodex Medical Systems, Shirley, NY, USA). Before dynamometry, participants completed a 10-min warm-up on a cycle ergometer. Participants then received standardized verbal instructions and were familiarized with the required knee-extension and knee-flexion efforts before recorded trials began. Participants were seated according to the manufacturer’s recommendations. The trunk, pelvis, and tested thigh were stabilized using straps, and the distal lower leg was secured to the dynamometer lever arm using a padded malleolus strap. Participants were instructed to hold the stabilization handles throughout all maximal efforts. Gravity correction was applied according to the manufacturer’s procedures before testing. The final analysis used the Biodex-reported peak torque value, labelled in the Biodex report as Szczyt. moment siły (N·m), corresponding to the best repetition identified by the software rather than the average of repeated attempts.

Testing was performed in a fixed order for all participants with respect to contraction mode, angle, and velocity. Isometric testing was completed first at 10°, 30°, 60°, 90°, and 110° of knee flexion. At each angle, participants performed repeated maximal voluntary knee-extension and knee-flexion contractions lasting 5 s, with 20 s of rest between contractions and 1 min of rest between angles. The isometric angle order was the same for all participants and was not randomized.

Isokinetic testing was then performed using concentric knee extension and concentric knee flexion at 60°/s, 150°/s, and 300°/s in a fixed ascending velocity order. Five maximal repetitions were performed at each velocity, with approximately 20 s of rest between velocity conditions. The Biodex summary report retained peak torque, the repetition in which peak torque occurred, peak-torque angle, coefficient of variation, work, power, range of motion, and agonist/antagonist ratio. The limb-testing order was not randomized and was not consistently retained in the analysis records. For angle-matched data reduction, flexor and extensor torque values were extracted from the exported torque-angle data at the same knee joint angles used in the isometric protocol (10°, 30°, 60°, 90°, and 110°). The tested variables were knee extensor torque, knee flexor torque, and the angle-specific H/Q ratio for each participant, limb, angle, and contraction mode. Individual repetition-level torque values were not retained in the final analysis file.

### 2.3. Angle-Specific H/Q Ratio

The angle-specific H/Q ratio was calculated as the torque produced by the knee flexors divided by the torque produced by the knee extensors at the same joint angle and limb:

H/Q ratio = flexor torque at angle *θ*/extensor torque at angle* θ*

Ratios were calculated separately for each participant, limb, contraction mode, and joint angle. The H/Q ratio was interpreted as a descriptive measure of relative knee flexor-to-extensor torque balance and not as an independent indicator of injury risk. Because ratio outcomes can be distorted by small denominator values, ratio results were interpreted together with the underlying extensor and flexor torques, especially at endpoint angles.

### 2.4. Statistical Analysis

All statistical analyses were performed on the cleaned repeated-measures dataset and analyses were performed using IBM SPSS Statistics for Windows, version 27.0 (IBM Corp., Armonk, NY, USA). H/Q ratios were recalculated from raw flexor and extensor torque values and cross-checked against the exported H/Q-ratio file. Most recalculated and exported values matched within rounding error. Four large non-rounding discrepancies were identified, all at the 10° endpoint angle, where reconstructed raw-derived ratios produced implausible values because of near-zero extensor denominator values or numerator-denominator mismatch during reconstruction of the analysis file. For these four observations, the exported H/Q-ratio file was retained as the reference value for the ratio analysis. The four corrected observations are reported in Supplementary Table S1 with anonymized case labels, limb, mode, angle, torque-derived ratio, exported ratio, final value used, and correction rationale.

Because H/Q ratios were right-skewed and sensitive to small extensor torque values, inferential statistics were performed on log-transformed H/Q ratios. The primary analysis used a three-factor repeated-measures ANOVA with joint angle (10°, 30°, 60°, 90°, and 110°), contraction mode (isometric, 60°/s, 150°/s, and 300°/s), and limb (left and right) as within-subject factors. Model assumptions were evaluated by inspecting residual distributions and by checking the repeated-measures structure for sphericity-related concerns. The Results report exact degrees of freedom, F statistics, *p*-values, partial eta squared, and approximate 95% confidence intervals for partial eta squared where available.

Operational endpoint instability was defined for interpretation as increased ratio dispersion and evidence of denominator sensitivity, assessed using H/Q-ratio variability, range, frequency of extreme ratios, and the frequency of small extensor-torque values. A sensitivity analysis was performed by repeating the repeated-measures ANOVA after removing endpoint angles and retaining only 30°, 60°, and 90°. This analysis was used to evaluate whether the main findings were driven by unstable endpoint ratios at 10° and 110°.

Where interaction effects were observed, paired post-hoc comparisons were used to compare contraction modes at matched angles and limbs. Holm correction was applied within each angle-by-limb family, and a more conservative global Holm interpretation was also considered to avoid overemphasis of isolated pairwise findings. Statistical significance was set at *p* < 0.05. All analyses were interpreted cautiously because the study was exploratory and the sample was small.

## 3. Results

### 3.1. Torque-Angle Descriptive Pattern

Across the tested range of motion, extensor torque generally increased from near extension toward the mid-to-flexed range, whereas flexor torque showed a different joint-angle profile. In isometric conditions, extensor peak torque occurred at approximately 90°, whereas flexor peak torque occurred at approximately 30°. Under isokinetic conditions, the angle at which peak extensor torque occurred shifted toward smaller angles as velocity increased. The peak-torque angles are summarized in Table 2.

### 3.2. Descriptive H/Q Ratio Profiles

**Figure 1:**
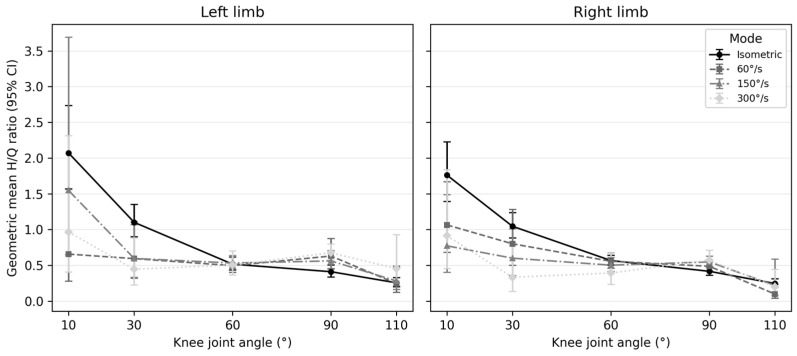
Geometrics mean H/Q ratio with 95% confidence intervals by joint angle, contraction mode, and limb. H/Q = hamstring-to-quadriceps ratio.

Angle-specific H/Q ratios showed clear joint-angle-dependent behavior. Isometric ratios were highest near 10° and decreased progressively toward 110°, whereas isokinetic ratios showed more variable profiles, particularly at the endpoint angles. The main descriptive ratios are presented as geometric means with 95% confidence intervals in Table 3 and visualized in Figure 1. To keep the Results section focused, the detailed endpoint-stability and absolute-torque diagnostics are reported in Supplementary Table S2.

### 3.3. Endpoint Diagnostics and Sensitivity Rationale

Endpoint diagnostics supported the decision to examine central angles separately from the full angle set. When values were pooled across limbs and contraction modes, dispersion was greatest at 10° and 110°. At 10°, the H/Q ratio ranged from 0.04 to 10.98, the coefficient of variation was 101.9%, 22 of 112 observations exceeded H/Q > 3, and 60 of 112 extensor-torque values were <10 Nm. At 110°, the H/Q ratio ranged from 0.00 to 5.71, the coefficient of variation was 162.6%, and 29 of 112 extensor-torque values were <10 Nm. By contrast, 60° and 90° showed lower dispersion and fewer small-denominator observations. The 30° angle also showed high relative dispersion, but it had no extensor-torque values < 10 Nm and far fewer extreme ratios than 10°. These diagnostics indicate that endpoint ratios were more sensitive to denominator behavior than central-angle ratios. Detailed endpoint-stability statistics and the corresponding absolute extensor and flexor torque descriptors are provided in Supplementary Table S2.

### 3.4. Repeated-Measures Analysis of Log-Transformed H/Q Ratio

**Figure 2:**
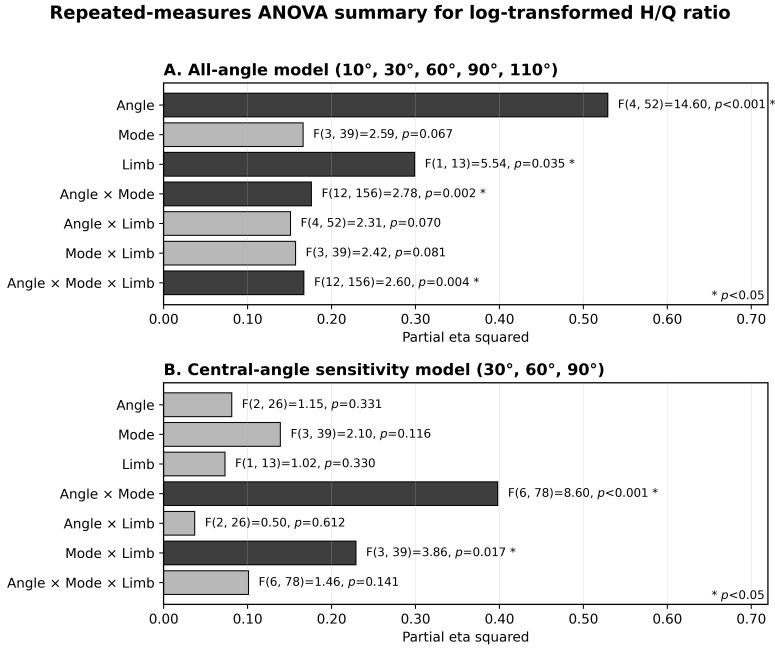
Visual summary of repeated-measures ANOVA results for log-transformed H/Q ratios. Bars show partial eta squared for each within-subject effect in the all-angle model (**A**) and central-angle sensitivity model (**B**). Dark bars indicate *p* < 0.05. The central-angle model retained only 30°, 60°, and 90°.

The primary repeated-measures ANOVA using all five joint angles demonstrated a significant main effect of angle on log-transformed H/Q ratio (F(4,52) = 14.60, *p* < 0.001, partial η^2^ = 0.529, approximate 95% CI for partial η^2^ = 0.297–0.632). The main effect of contraction mode approached statistical significance (F(3,39) = 2.59, *p* = 0.067, partial η^2^ = 0.166, 95% CI = 0.000–0.325), and there was a significant limb effect (F(1,13) = 5.54, *p* = 0.035, partial η^2^ = 0.299, 95% CI = 0.000–0.571). Importantly, the angle × mode interaction was significant (F(12,156) = 2.78, *p* = 0.002, partial η^2^ = 0.176, 95% CI = 0.028–0.223), indicating that differences between contraction modes depended on joint angle. The three-way angle × mode × limb interaction was also significant (F(12,156) = 2.60, *p* = 0.004, partial η^2^ = 0.167, 95% CI = 0.021–0.212)(Figure 2). The complete ANOVA output, including non-significant effects and central-angle sensitivity results, is provided in Supplementary Table S3.

### 3.5. Central-Angle Sensitivity Analysis and Post-Hoc Findings

When endpoint angles were removed and only 30°, 60°, and 90° were retained, the angle × mode interaction remained significant (F(6,78) = 8.60, *p* < 0.001, partial η^2^ = 0.398, approximate 95% CI = 0.189–0.495). In contrast, the main effect of angle was no longer significant (F(2,26) = 1.15, *p* = 0.331). These findings suggest that the strong main angle effect in the all-angle model was partly influenced by endpoint behavior, whereas mode-dependent differences at matched angles remained evident in the central-angle analysis. This pattern supports the use of the central-angle model as a robustness check, but it does not justify excluding endpoint values from descriptive reporting; instead, endpoint ratios should be presented together with the underlying flexor and extensor torques.

**Figure 3:**
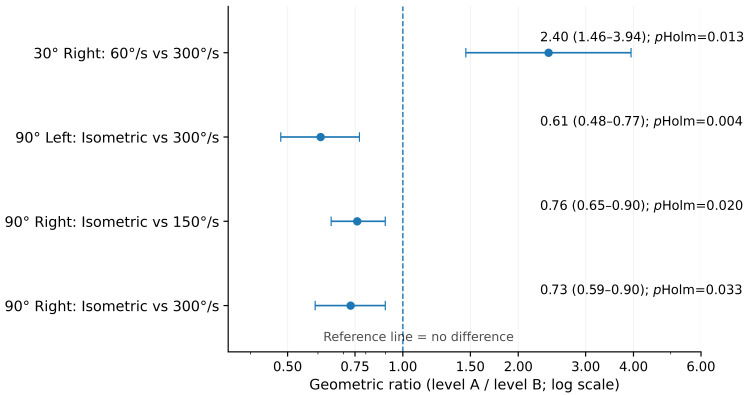
Forest plot of significant post-hoc contraction-mode comparisons within matched angle-by-limb families. Points represent geometric ratios after back-transformation from the log scale, and horizontal bars indicate 95% confidence intervals. Values above 1 indicate that level A was higher than level B; the dashed vertical line indicates no difference.

Post-hoc comparisons identified selected contraction-mode differences at matched joint angles and limbs. Using Holm correction within each angle-by-limb family, significant differences were found at 30° in the right limb, where the 60°/s ratio exceeded the 300°/s ratio (geometric ratio = 2.40, 95% CI = 1.46–3.94, pHolm = 0.013), and at 90°, where selected isokinetic ratios differed from isometric ratios: 90° left isometric vs 300°/s (geometric ratio = 0.61, 95% CI = 0.48–0.77, pHolm = 0.004), 90° right isometric vs 150°/s (geometric ratio = 0.76, 95% CI = 0.65–0.90, pHolm = 0.020), and 90° right isometric vs 300°/s (geometric ratio = 0.73, 95% CI = 0.59–0.90, pHolm = 0.033). Under the more conservative global Holm correction, only the 90° left-limb comparison between isometric and 300°/s remained significant (global pHolm = 0.037). Therefore, interpretation should focus on the overall angle × mode interaction and the central-angle sensitivity result rather than on isolated pairwise contrasts (Figure 3). The complete set of pairwise comparisons, including non-significant results, is provided as Supplementary Table S2.

## 4. Discussion

The present exploratory study examined whether angle-matched hamstring-to-quadriceps (H/Q) ratios derived from isometric and isokinetic dynamometry can be interpreted as directly interchangeable. The principal finding was that H/Q ratios depended on knee joint angle and that the effect of contraction mode varied across joint positions. The significant angle × mode interaction in both the all-angle and central-angle analyses indicates that the numerical value and interpretation of the H/Q ratio depend not only on relative flexor and extensor torque capacity, but also on the mechanical context in which the ratio is calculated. This supports the central methodological interpretation of the study: isometric and isokinetic H/Q ratios should not be treated as equivalent simply because they are expressed using the same flexor/extensor formula.

A second important finding was that endpoint angles showed greater instability than central angles. At 10°, the H/Q ratio had a CV of 101.9% and 22/112 observations exceeded H/Q > 3, while 60/112 extensor torque values were <10 Nm. At 110°, the H/Q ratio had a CV of 162.6%, and 29/112 extensor torque values were <10 Nm. In contrast, the 60° angle showed lower ratio dispersion (CV = 32.6%) and no H/Q values > 2 or >3. This pattern indicates that endpoint behavior contributed to the overall angle effect, while mode-dependent differences were still present at more mechanically stable central angles. Endpoint findings should therefore be interpreted as methodological observations about ratio stability rather than as clinical threshold evidence.

The present findings are consistent with previous work showing that H/Q ratios are influenced by contraction mode, angular velocity, gravity correction, joint position, and the method used to define the ratio [12,13,14,15,16]. They also align with angle-specific studies in athletic and anterior cruciate ligament populations, where strength deficits, normative profiles, and H/Q-ratio patterns vary across the knee-flexion range and may not be fully captured by peak-torque metrics alone [20,21,22,23,24,25,26]. The present study refines this literature by applying an angle-matched comparison across isometric and isokinetic modes in the same participants. The results support previous evidence that H/Q interpretation is angle- and velocity-dependent, but they do not support broad statements that one contraction mode or velocity yields consistently higher or lower ratios across all angles and limbs.

### 4.1. Angle-Specific Behavior of the H/Q Ratio

The angle-specific behavior observed in this study is biomechanically plausible. Knee extensor and flexor torques do not change identically across the range of motion. Differences in muscle length-tension behavior, tendon properties, moment arm geometry, and neural activation strategy mean that the quadriceps and hamstrings can reach their highest torques at different joint angles [27,28,29,30]. When two angle-dependent torque curves are converted into a ratio, even small differences in the shapes of those curves can create substantial changes in the H/Q value. This is especially important when the denominator torque is small, because ratio inflation may occur without a corresponding increase in flexor torque capacity.

This is particularly important because the H/Q ratio is often discussed as a single descriptor of knee muscle balance. A single ratio value may be useful as a summary statistic, but it cannot represent the full angle-dependent relationship between flexor and extensor torque. For example, a ratio obtained close to knee extension may reflect a very different mechanical situation from a ratio obtained at 60° or 90° of knee flexion. The same numerical ratio at two different angles may therefore have different meanings if the underlying flexor and extensor torques, muscle lengths, and lever arms differ.

The current data support the value of angle-specific H/Q analysis as a descriptive approach. However, they also show that angle-specific results must be reported together with contraction mode and absolute torque. The angle × mode interaction remained significant after 10° and 110° were removed, indicating that the non-interchangeability of isometric and isokinetic ratios was not explained only by unstable endpoint values. In practical terms, an H/Q result should be described by at least four pieces of information: the joint angle, contraction mode or angular velocity, limb, and underlying flexor and extensor torque values.

### 4.2. Endpoint Instability and Denominator Effects

Endpoint behavior was one of the clearest methodological issues in the present analysis. H/Q ratios were especially variable near 10° and 110°, and several endpoint values were sensitive to low extensor torque. This is a common limitation of ratio outcomes. Because the extensor torque is the denominator, a small extensor value can produce a large H/Q ratio even when flexor torque is not unusually high. In such cases, an apparently elevated ratio may reflect denominator instability rather than a meaningful increase in relative hamstring capacity. The added absolute-torque analysis makes this distinction explicit.

This problem is particularly relevant in dynamometry because torque measurement near the limits of the tested range may be influenced by mechanical leverage, participant comfort, stabilization quality, acceleration and deceleration phases, and the ability to produce maximal effort at an end-range position. Isokinetic testing is especially sensitive to these issues because the limb must accelerate into and decelerate out of the movement range, and torque values at the extremes may not reflect the same mechanical conditions as values obtained at central joint angles [3].

The central-angle sensitivity analysis is therefore important. By removing 10° and 110°, the analysis reduced the influence of the most unstable positions. The disappearance of the main angle effect in this model suggests that endpoint values contributed substantially to the apparent overall angle dependence. However, the persistence of the angle × mode interaction indicates that mode-dependent differences remained even when the analysis was restricted to more stable central positions. This strengthens the conclusion that the testing mode itself matters, while also supporting cautious interpretation of endpoint ratios.

For applied use, endpoint ratios should not be interpreted in isolation. They may provide descriptive information when end-range strength is clinically relevant, but they should be presented together with the raw flexor and extensor torques and with a clear denominator-stability check. Reporting only the ratio can hide whether a high value is caused by strong flexor torque, weak extensor torque, or both. This is particularly important in small samples, clinical populations, or longitudinal monitoring where individual values can strongly influence group-level summaries.

### 4.3. Influence of Contraction Mode and Velocity

Isometric and isokinetic testing impose different mechanical and neuromuscular demands. Isometric testing measures force production at a fixed joint position, without visible joint movement. It is therefore highly angle-specific and may be useful when the goal is to characterize force capacity at predefined positions [5,6,7]. Isokinetic testing, in contrast, measures torque during movement at a controlled angular velocity and allows torque to be examined across a continuous range of motion [3,4]. Because these modes differ in joint movement, contraction velocity, and stabilization demands, their H/Q ratios should not be assumed to reflect the same construct.

In the present study, the main effect of contraction mode only approached statistical significance, but the angle × mode interaction was significant. This distinction is important. It indicates that the question is not simply whether isometric testing produces higher or lower H/Q ratios than isokinetic testing overall. Instead, the effect of mode depends on the knee angle at which the comparison is made. A general statement such as “isokinetic ratios are higher than isometric ratios” would therefore be incomplete unless the angle and velocity are specified.

The descriptive findings support this interpretation. Isometric ratios were highest near 10° and decreased toward greater knee flexion angles, whereas the isokinetic profiles showed more variable behavior across velocities and endpoint positions. These differences likely reflect the combined influence of force-length properties, force-velocity constraints, moment arm changes, and the angle at which each muscle group can produce torque most effectively. Previous studies have similarly reported that H/Q ratios vary with angular velocity and contraction mode [12,13,16].

This helps explain why conventional peak-torque H/Q ratios can be difficult to compare across studies. Peak extensor torque and peak flexor torque may occur at different angles, and the angle at which peak torque occurs may shift as velocity changes. When a ratio is calculated from peak values that do not occur at the same joint position, the result combines strength capacity with measurement geometry. Angle-matched analysis makes this dependency more visible and may reduce one source of ambiguity in H/Q interpretation.

### 4.4. Interpretation of Post-Hoc Findings

The post-hoc comparisons identified selected contraction-mode differences within matched angle-by-limb families. However, these pairwise results should be interpreted cautiously. After within-family Holm correction, only four comparisons remained significant, and under the more conservative global Holm interpretation only the 90° left-limb isometric versus 300°/s comparison remained significant. Therefore, the main inference should come from the repeated-measures interaction and sensitivity analysis rather than from isolated pairwise contrasts. The complete post-hoc table should be used to show the full pattern rather than selectively emphasizing significant comparisons.

This conservative interpretation is important because the study was exploratory and the sample was small. In repeated-measures designs with many angle, mode, and limb combinations, isolated significant post-hoc findings can occur even when the broader pattern is the more meaningful result. The present data therefore support the conclusion that H/Q ratios behave differently across angles and modes, but they do not justify strong claims about one specific velocity or limb being consistently superior or inferior across all positions.

The limb effect also requires cautious interpretation. The primary model identified a significant left-right limb effect, but the study was not designed to test dominance-related asymmetry, sport-specific loading, or clinical side-to-side deficits. Dominant limb was right in eight participants and left in six participants; however, the sample was too small for a dominance-stratified analysis. The limb finding should therefore be treated as descriptive. Future studies should include prespecified dominant/non-dominant classification, training-history stratification, and sufficient sample size to examine whether angle-specific H/Q patterns differ by dominance, sport exposure, or previous injury history.

### 4.5. Practical Implications for Research and Applied Testing

For researchers, the present findings emphasize that H/Q ratios should be reported with their measurement context. At minimum, authors should state whether the ratio is conventional, functional, mixed, peak-torque based, or angle-specific; whether it was obtained during isometric or isokinetic testing; the joint angle or velocity used; the limb; and the underlying flexor and extensor torque values. This level of detail is necessary because the same H/Q formula can produce values with different biomechanical meanings depending on the testing protocol [14,15]. For future angle-specific studies, central angles such as 30°, 60°, and 90° may provide more interpretable monitoring points than endpoint angles, but this recommendation should be confirmed by protocol-specific test-retest reliability, because reliability can differ between peak torque, ratio outcomes, and angle-specific values [9,10,11,26].

For clinicians and sport practitioners, the most defensible recommendation is to avoid substituting isometric and isokinetic H/Q ratios for one another during monitoring. If an athlete or patient is followed over time, the same angle, contraction mode, limb, warm-up, stabilization, and analysis method should be retained across sessions. Angle-specific ratios should be interpreted alongside absolute knee-flexor and knee-extensor torque, limb-symmetry indices, symptoms, sport demands, and rehabilitation phase. Endpoint ratios may be useful for specific clinical questions about end-range function, but they should not be interpreted without confirming whether the denominator torque is stable.

The findings also support a cautious interpretation of H/Q ratios in injury-risk discussions. Some prospective evidence has linked strength imbalance with hamstring injury risk, but other cohort and systematic-review evidence suggests that H/Q ratio alone has limited value as an independent predictor of hamstring or anterior cruciate ligament injury [17,18,19]. The H/Q ratio describes relative torque production, but it does not directly capture muscle activation timing, fatigue, tissue capacity, coordination strategy, running mechanics, or sport exposure. It should therefore be considered one component of a broader neuromuscular assessment rather than a stand-alone screening tool.

From a reporting perspective, geometric means and log-transformed analyses were useful because the ratio data were right-skewed and sensitive to small denominator values. This approach reduced the influence of extreme values and provided a more appropriate framework for inference. Future studies using H/Q ratios should consider similar transformations or robust statistical methods when ratio distributions are skewed.

### 4.6. Limitations

Several limitations should be acknowledged. First, the sample was small and no a priori power analysis was performed before data collection. The study was therefore underpowered for small or moderate interaction effects and should be interpreted as exploratory. The repeated-measures design increased the number of within-participant observations, but the number of independent participants remained 14. Second, the sample consisted of healthy young adults and was not large enough for sex-specific, sport-specific, or dominance-specific analyses. Dominant limb was recorded descriptively, but detailed training volume, years of participation, and competition level were not recorded using a validated instrument. Third, participants were apparently healthy and did not represent injured athletes, post-operative patients, older adults, or individuals undergoing rehabilitation. The results should therefore be interpreted as methodological and exploratory rather than directly clinical.

Fourth, the study did not measure electromyography, muscle architecture, tendon stiffness, or muscle-tendon behavior. Therefore, the mechanisms underlying the observed angle-specific and mode-dependent differences cannot be directly confirmed. The results are consistent with known torque-angle and torque-velocity principles, but they do not determine whether the differences were driven primarily by mechanical leverage, neural activation, muscle length, or participant-specific strategy.

Fifth, endpoint ratios were highly variable and appeared sensitive to low extensor torque. Although this was an important methodological finding, it also means that endpoint results should be interpreted cautiously and together with absolute torque values. Sixth, the testing order was fixed for contraction mode, joint angle, and angular velocity, and the limb-testing order was not randomized or consistently retained in the analysis records. Therefore, possible order, fatigue, or learning effects cannot be excluded. This limitation is relevant because the protocol involved multiple maximal contractions across angles and velocities. Seventh, protocol-specific test-retest reliability was not established. The Biodex summary report provided condition-level coefficient-of-variation values for repeated contractions, but individual repetition-level torque values were not retained in the final analysis file. Therefore, condition-specific ICCs and smallest detectable changes could not be calculated retrospectively. Literature-based reliability evidence supports the general dynamometry approach and the reproducibility of many peak-torque outcomes [8,9,10,11], but it does not replace reliability estimation for the present angle-matched H/Q protocol.

Finally, the analysis focused primarily on ratios rather than absolute torque capacity. Ratios can be useful for describing relative balance, but they can obscure whether differences are caused by the numerator, denominator, or both. For this reason, endpoint and angle-specific H/Q ratios should be interpreted together with the underlying knee flexor and extensor torques.

### 4.7. Future Directions

Future studies should first establish protocol-specific test-retest reliability for angle-matched isometric and isokinetic H/Q ratios, including ICC, coefficient of variation, standard error of measurement, and smallest detectable change at each angle, limb, and contraction mode. Reliability should be reported separately for central angles and endpoint angles because endpoint denominator effects may reduce interpretability even when peak torque reliability is acceptable [10,11]. Future protocols should also randomize or counterbalance limb order, angle order, and contraction-mode order when feasible.

Second, prospective studies should combine angle-specific H/Q ratios with absolute torque, electromyography, muscle architecture or imaging, fatigue measures, and sport-specific movement assessments. These designs would help determine whether angle-specific ratio changes reflect neural activation, muscle-tendon mechanics, denominator behavior, or genuine changes in flexor/extensor balance. Third, longitudinal studies in training, rehabilitation, and return-to-sport settings are needed to test whether angle-specific ratios are responsive to intervention and whether they provide information beyond conventional peak-torque ratios and normative angle-specific torque profiles [20,21,22,23,24,25,26].

Finally, larger studies should include clearly defined physical activity status, sport participation, training history, sex, limb dominance, and previous injury status. These variables are necessary to determine whether angle-specific H/Q profiles differ meaningfully between athletes, clinical groups, and healthy controls.

## 5. Conclusions

In this exploratory repeated-measures study of 14 healthy young adults, angle-specific H/Q ratios were influenced by joint position and showed contraction-mode-dependent behavior at matched knee angles. The significant angle × mode interaction indicates that isometric and isokinetic H/Q ratios should not be treated as directly interchangeable. Endpoint angles showed greater variability and should be interpreted together with the underlying flexor and extensor torque values because small extensor denominator values can distort ratio estimates. These findings support more transparent reporting of joint angle, contraction mode, limb, absolute torque, and ratio-stability information in H/Q assessment. Larger studies with formal reliability testing, complete participant characterization, and longitudinal designs are required before clinical monitoring thresholds or injury-risk interpretations can be proposed.

## Figures and Tables

**Table 2:** Joint angles at which peak torque occurred for knee extensors (EX) and flexors (FX) in the left (L) and right (R) limbs.

Contraction Mode	EX L (°)	EX R (°)	FX L (°)	FX R (°)
60°/s	71.0	71.5	52.0	51.0
150°/s	65.5	67.5	54.5	60.5
300°/s	61.0	61.5	44.0	43.0
Isometric	90.0	90.0	30.0	30.0

**Table 3:** Geometric mean angle-specific H/Q ratios with 95% confidence intervals.

Mode	Angle (°)	Left GM (95% CI)	Right GM (95% CI)
Isometric	10	2.07 (1.57–2.73)	1.76 (1.39–2.23)
Isometric	30	1.10 (0.90–1.35)	1.05 (0.88–1.24)
Isometric	60	0.52 (0.43–0.61)	0.57 (0.50–0.64)
Isometric	90	0.41 (0.34–0.50)	0.42 (0.36–0.48)
Isometric	110	0.26 (0.20–0.33)	0.25 (0.20–0.31)
60°/s	10	0.66 (0.28–1.56)	1.06 (0.68–1.67)
60°/s	30	0.59 (0.32–1.11)	0.80 (0.50–1.28)
60°/s	60	0.50 (0.40–0.61)	0.56 (0.46–0.67)
60°/s	90	0.63 (0.45–0.87)	0.49 (0.42–0.56)
60°/s	110	0.24 (0.12–0.49)	0.10 (0.04–0.24)
150°/s	10	1.55 (0.65–3.69)	0.77 (0.40–1.49)
150°/s	30	0.60 (0.33–1.07)	0.60 (0.36–1.01)
150°/s	60	0.53 (0.45–0.64)	0.50 (0.41–0.61)
150°/s	90	0.56 (0.45–0.71)	0.55 (0.48–0.63)
150°/s	110	0.28 (0.17–0.48)	0.22 (0.08–0.59)
300°/s	10	0.97 (0.40–2.31)	0.91 (0.45–1.84)
300°/s	30	0.45 (0.23–0.88)	0.33 (0.14–0.82)
300°/s	60	0.50 (0.36–0.70)	0.39 (0.23–0.67)
300°/s	90	0.68 (0.57–0.80)	0.57 (0.46–0.71)
300°/s	110	0.45 (0.22–0.93)	0.20 (0.09–0.44)

GM = geometric mean. Confidence intervals were calculated on the log scale and back-transformed. Values are descriptive and should be interpreted cautiously at endpoint angles because small extensor torque values can inflate ratios.

## Data Availability

The data presented in this study are available from the corresponding author upon reasonable request, subject to privacy and ethical restrictions.

## Supplementary Materials

The following supporting information can be downloaded at: https://www.mdpi.com/article/10.3390/bioengineering13070744/s1, Table S1: Corrected H/Q-ratio discrepancies. Table S2: All post-hoc contraction-mode comparisons. Table S3: Endpoint-stability and absolute torque descriptors. Table S4: Complete repeated-measures ANOVA output for log-transformed H/Q ratios. Table S1 documents the four corrected H/Q-ratio discrepancies; Table S2 reports endpoint-stability and absolute-torque descriptors; Table S3 provides the complete repeated-measures ANOVA output; Table S4 reports all pairwise contraction-mode comparisons.

## Abbreviations

The following abbreviations are used in this manuscript:

**Abbreviation**	**Definition**
ANOVA	Analysis of variance
AST	Angle-specific torque
EX	Knee extension/extensors
FX	Knee flexion/flexors
H/Q	Hamstring-to-quadriceps
L	Left
R	Right
ROM	Range of motion
SD	Standard deviation
