# Supplementary material for: Angle-Matched Isometric and Isokinetic Hamstring-to-Quadriceps Ratios Are Not Directly Interchangeable: An Exploratory Multi-Angle Dynamometry Study
Source: Bioengineering (Basel). 2026 Jun 26;13(7):744. doi: 10.3390/bioengineering13070744 (PMC13405163; doi:10.3390/bioengineering13070744)
Supplement: Supplementary file 1 [file bioengineering-13-00744-s001.zip › bioengineering-4395706-supplementary.pdf]

Supplementary Tables

Supplementary material for bioengineering-4395706. All participant labels are anonymized.

Supplementary Table S1. Corrected H/Q-ratio discrepancies

Four large non-rounding discrepancies were identified during cross-checking of torque-derived and exported H/Q-ratio values. All occurred at 10°. The exported ratio-file value was retained for the final H/Q analysis.

CASE	LIMB	MODE	ANGLE (°)	EX TORQUE (NM)	FX TORQUE (NM)	RAW-DERIVED H/Q	EXPORTED H/Q	FINAL H/Q USED	RATIONALE
**CASE 1**	Right	60°/s	10	0.637	36.113	56.65	3.10	3.10	ratio-file correction for major raw-derived discrepancy
**CASE 2**	Left	150°/s	10	1.988	30.220	15.20	5.19	5.19	ratio-file correction for major raw-derived discrepancy
**CASE 3**	Right	60°/s	10	0.019	94.595	5037.01	1.25	1.25	ratio-file correction for major raw-derived discrepancy
**CASE 4**	Right	60°/s	10	0.800	13.600	17.00	1.70	1.70	ratio-file correction for major raw-derived discrepancy

EX = knee extensor torque; FX = knee flexor torque; H/Q = hamstring-to-quadriceps ratio.

Supplementary Table S2. All post-hoc contraction-mode comparisons

Paired comparisons were performed on log-transformed H/Q ratios within matched angle-by-limb families. Estimates are back-transformed geometric ratios. pHolm-family refers to Holm adjustment within each angle-by-limb family; pHolm-global refers to Holm adjustment across all post-hoc comparisons.

ANGLE	LIMB	COMPARISON	N	GEO. RATIO	95% CI	T	DF	P RAW	PHOLM-FAMILY	PHOLM-GLOBAL
**10**	Left	Isometric vs 60°/s	14	3.15	1.28–7.77	2.74	13	0.017	0.100	0.919
**10**	Left	Isometric vs 150°/s	14	1.34	0.53–3.40	0.68	13	0.511	0.876	1.000
**10**	Left	Isometric vs 300°/s	14	2.14	0.86–5.33	1.81	13	0.094	0.471	1.000
**10**	Left	60°/s vs 150°/s	14	0.42	0.14–1.30	-1.66	13	0.121	0.486	1.000
**10**	Left	60°/s vs 300°/s	14	0.68	0.24–1.92	-0.80	13	0.438	0.876	1.000
**10**	Left	150°/s vs 300°/s	14	1.60	0.80–3.21	1.46	13	0.168	0.503	1.000
**10**	Right	Isometric vs 60°/s	14	1.65	1.02–2.69	2.23	13	0.044	0.221	1.000
**10**	Right	Isometric vs 150°/s	14	2.28	1.14–4.55	2.58	13	0.023	0.138	1.000
**10**	Right	Isometric vs	14	1.93	0.90–	1.85	13	0.086	0.346	1.000

		300°/s			4.14					
**10**	Right	60°/s vs 150°/s	14	1.38	0.71–2.67	1.05	13	0.313	0.938	1.000
**10**	Right	60°/s vs 300°/s	14	1.17	0.56–2.42	0.45	13	0.658	1.000	1.000
**10**	Right	150°/s vs 300°/s	14	0.85	0.33–2.18	-0.38	13	0.709	1.000	1.000
**30**	Left	Isometric vs 60°/s	14	1.85	1.00–3.42	2.17	13	0.049	0.195	1.000
**30**	Left	Isometric vs 150°/s	14	1.85	1.05–3.24	2.35	13	0.035	0.175	1.000
**30**	Left	Isometric vs 300°/s	14	2.47	1.23–4.95	2.80	13	0.015	0.090	0.839
**30**	Left	60°/s vs 150°/s	14	1.00	0.67–1.49	-0.02	13	0.983	0.983	1.000
**30**	Left	60°/s vs 300°/s	14	1.33	0.86–2.07	1.40	13	0.186	0.372	1.000
**30**	Left	150°/s vs 300°/s	14	1.34	1.00–1.79	2.13	13	0.053	0.195	1.000
**30**	Right	Isometric vs 60°/s	14	1.30	0.79–2.15	1.15	13	0.270	0.270	1.000
**30**	Right	Isometric vs 150°/s	14	1.74	1.02–2.96	2.25	13	0.042	0.168	1.000
**30**	Right	Isometric vs 300°/s	14	3.13	1.25–7.88	2.68	13	0.019	0.095	1.000
**30**	Right	60°/s vs 150°/s	14	1.33	0.96–1.85	1.90	13	0.079	0.238	1.000
**30**	Right	60°/s vs 300°/s	14	2.40	1.46–3.94	3.83	13	0.002	0.013	0.124
**30**	Right	150°/s vs 300°/s	14	1.80	0.90–3.59	1.84	13	0.089	0.238	1.000
**60**	Left	Isometric vs 60°/s	14	1.04	0.81–1.34	0.34	13	0.737	1.000	1.000
**60**	Left	Isometric vs 150°/s	14	0.97	0.81–1.16	-0.38	13	0.712	1.000	1.000
**60**	Left	Isometric vs 300°/s	14	1.02	0.75–1.39	0.16	13	0.874	1.000	1.000
**60**	Left	60°/s vs 150°/s	14	0.93	0.77–1.13	-0.79	13	0.442	1.000	1.000
**60**	Left	60°/s vs 300°/s	14	0.98	0.84–1.15	-0.23	13	0.820	1.000	1.000
**60**	Left	150°/s vs 300°/s	14	1.06	0.82–1.36	0.45	13	0.658	1.000	1.000
**60**	Right	Isometric vs 60°/s	14	1.02	0.81–1.28	0.18	13	0.859	0.859	1.000
**60**	Right	Isometric vs 150°/s	14	1.13	0.96–1.33	1.58	13	0.139	0.694	1.000
**60**	Right	Isometric vs 300°/s	14	1.44	0.84–2.47	1.47	13	0.164	0.694	1.000
**60**	Right	60°/s vs 150°/s	14	1.11	0.92–1.33	1.20	13	0.250	0.750	1.000
**60**	Right	60°/s vs 300°/s	14	1.42	0.94–2.14	1.82	13	0.092	0.549	1.000
**60**	Right	150°/s vs 300°/s	14	1.28	0.80–2.04	1.13	13	0.279	0.750	1.000
**90**	Left	Isometric vs 60°/s	14	0.65	0.45–0.94	-2.49	13	0.027	0.109	1.000
**90**	Left	Isometric vs 150°/s	14	0.73	0.56–0.95	-2.60	13	0.022	0.109	1.000
**90**	Left	Isometric vs 300°/s	14	0.61	0.48–0.77	-4.48	13	<0.001	0.004	0.037
**90**	Left	60°/s vs 150°/s	14	1.12	0.78–1.60	0.69	13	0.502	1.000	1.000
**90**	Left	60°/s vs 300°/s	14	0.93	0.66–1.32	-0.43	13	0.672	1.000	1.000
**90**	Left	150°/s vs 300°/s	14	0.83	0.67–1.04	-1.80	13	0.094	0.283	1.000
**90**	Right	Isometric vs 60°/s	14	0.86	0.71–1.03	-1.78	13	0.099	0.397	1.000
**90**	Right	Isometric vs 150°/s	14	0.76	0.65–0.90	-3.59	13	0.003	0.020	0.189
**90**	Right	Isometric vs 300°/s	14	0.73	0.59–0.90	-3.23	13	0.007	0.033	0.374
**90**	Right	60°/s vs 150°/s	14	0.89	0.77–1.03	-1.77	13	0.100	0.397	1.000
**90**	Right	60°/s vs 300°/s	14	0.85	0.70–1.04	-1.77	13	0.100	0.397	1.000
**90**	Right	150°/s vs 300°/s	14	0.96	0.81–1.12	-0.60	13	0.559	0.559	1.000
**110**	Left	Isometric vs	14	1.04	0.52–	0.14	13	0.895	1.000	1.000

		60°/s			2.10					
**110**	Left	Isometric vs 150°/s	14	0.91	0.48–1.72	-0.33	13	0.749	1.000	1.000
**110**	Left	Isometric vs 300°/s	14	0.56	0.27–1.16	-1.71	13	0.111	0.663	1.000
**110**	Left	60°/s vs 150°/s	14	0.87	0.39–1.94	-0.38	13	0.712	1.000	1.000
**110**	Left	60°/s vs 300°/s	14	0.54	0.23–1.28	-1.54	13	0.147	0.728	1.000
**110**	Left	150°/s vs 300°/s	14	0.62	0.32–1.21	-1.55	13	0.146	0.728	1.000
**110**	Right	Isometric vs 60°/s	14	2.49	1.10–5.66	2.40	13	0.032	0.191	1.000
**110**	Right	Isometric vs 150°/s	14	1.12	0.43–2.90	0.26	13	0.800	1.000	1.000
**110**	Right	Isometric vs 300°/s	14	1.23	0.50–3.00	0.50	13	0.624	1.000	1.000
**110**	Right	60°/s vs 150°/s	14	0.45	0.15–1.36	-1.56	13	0.143	0.715	1.000
**110**	Right	60°/s vs 300°/s	14	0.49	0.17–1.41	-1.46	13	0.169	0.715	1.000
**110**	Right	150°/s vs 300°/s	14	1.10	0.39–3.12	0.19	13	0.850	1.000	1.000

Supplementary Table S3. Endpoint-stability and absolute torque descriptors

These descriptors quantify the dispersion of the H/Q ratio and the frequency of small extensor denominator values at each angle.

ANGLE (°)	N	H/Q MEAN ± SD	CV (%)	H/Q RANGE	H/Q >2	H/Q >3	EX <5 NM	EX <10 NM
**10**	112	1.90 ± 1.94	101.9	0.04–10.98	36	22	53	60
**30**	112	0.93 ± 0.96	103.1	0.01–7.83	4	2	0	0
**60**	112	0.55 ± 0.18	32.6	0.04–1.01	0	0	0	0
**90**	112	0.58 ± 0.30	52.2	0.24–2.76	1	0	1	3
**110**	112	0.46 ± 0.75	162.6	0.00–5.71	4	2	20	29

Absolute torque descriptors:

ANGLE (°)	EX MEDIAN [IQR] NM	EX RANGE NM	FX MEDIAN [IQR] NM	FX RANGE NM	EX <5 NM	EX <10 NM
**10**	5.28 [1.66–41.25]	0.02–115.70	11.21 [1.81–61.60]	0.05–191.70	53	60
**30**	79.48 [47.58–110.05]	11.30–158.20	63.21 [33.16–98.00]	0.12–179.90	0	0
**60**	126.91 [82.25–173.26]	16.07–264.90	73.80 [45.67–99.91]	0.65–162.70	0	0
**90**	108.15 [66.12–173.22]	4.43–361.60	58.30 [35.27–80.75]	5.84–141.30	1	3
**110**	37.97 [9.79–119.31]	0.43–319.40	5.36 [1.97–24.12]	0.18–125.30	20	29

Supplementary Table S4. Complete repeated-measures ANOVA output for log-transformed H/Q ratios.

Approximate 95% confidence intervals for partial η^2 were derived from the noncentral F distribution and should be interpreted cautiously because of the small sample size. The central-angle model retained only 30°, 60°, and 90°.

MODEL	EFFECT	DF	F	P	PARTIAL Η²	APPROX. 95% CI
**ALL ANGLES**	Angle	4, 52	14.60	<0.001	0.529	0.297–0.632
**ALL ANGLES**	Mode	3, 39	2.59	0.067	0.166	0.000–0.325
**ALL ANGLES**	Limb	1, 13	5.54	0.035	0.299	0.000–0.571
**ALL ANGLES**	Angle × Mode	12, 156	2.78	0.002	0.176	0.028–0.223
**ALL ANGLES**	Angle × Limb	4, 52	2.31	0.071	0.151	0.000–0.278
**ALL ANGLES**	Mode × Limb	3, 39	2.42	0.081	0.157	0.000–0.315
**ALL ANGLES**	Angle × Mode × Limb	12, 156	2.60	0.004	0.167	0.021–0.212
**CENTRAL ANGLES**	Angle	2, 26	1.15	0.331	0.081	0.000–0.271
**CENTRAL ANGLES**	Mode	3, 39	2.10	0.116	0.139	0.000–0.294
**CENTRAL ANGLES**	Limb	1, 13	1.02	0.330	0.073	0.000–0.373
**CENTRAL ANGLES**	Angle × Mode	6, 78	8.60	<0.001	0.398	0.189–0.495
**CENTRAL ANGLES**	Angle × Limb	2, 26	0.50	0.612	0.037	0.000–0.195
**CENTRAL ANGLES**	Mode × Limb	3, 39	3.86	0.017	0.229	0.008–0.391
**CENTRAL ANGLES**	Angle × Mode × Limb	6, 78	1.46	0.141	0.101	0.000–0.176
